# Tacrolimus Loaded Cationic Liposomes for Dry Eye Treatment

**DOI:** 10.3389/fphar.2022.838168

**Published:** 2022-02-04

**Authors:** Xiang Chen, Jicheng Wu, Xueqi Lin, Xingdi Wu, Xuewen Yu, Ben Wang, Wen Xu

**Affiliations:** ^1^ Eye Center, The Second Affiliated Hospital of Zhejiang University School of Medicine, Hangzhou, China; ^2^ Cancer Institute (Key Laboratory of Cancer Prevention and Intervention, China National Ministry of Education), The Second Affiliated Hospital, Zhejiang University School of Medicine, Hangzhou, China; ^3^ Institute of Translational Medicine, Zhejiang University, Hangzhou, China

**Keywords:** cationic liposome, drug delivery system, tacrolimus, dry eye, ocular bioavailability

## Abstract

Eye drops are ophthalmic formulations routinely used to treat dry eye. However, the low ocular bioavailability is an obvious drawback of eye drops owing to short ocular retention time and weak permeability of the cornea. Herein, to improve the ocular bioavailability of eye drops, a cationic liposome eye drop was constructed and used to treat dry eye. Tacrolimus liposomes exhibit a diameter of around 300 nm and a surface charge of +30 mV. Cationic liposomes could interact with the anionic ocular surface, extending the ocular retention time and improving tacrolimus amount into the cornea. The cationic liposomes notably prolonged the ocular retention time of eye drops, leading to an increased tacrolimus concentration in the ocular surface. The tacrolimus liposomes were also demonstrated to reduce reactive oxygen species and dry eye–related inflammation factors. The use of drug-loaded cationic liposomes is a good formulation in the treatment of ocular disease; the improved ocular retention time and biocompatibility give tremendous scope for application in the treatment of ocular disease, with further work in the area recommended.

## 1 Introduction

Eye drops are pretty restricted by low ocular bioavailability. Despite this, many clinicians prefer topical ophthalmic solutions to other treatment options as eye drops are accessible and noninvasive, and this formulation accounts for nearly 90% of ophthalmic drugs (Weng et al., 2018; Jain et al., 2019). Prolonging ocular retention time and prompting the ability to permeate into the cornea are two primary approaches to increase ocular bioavailability. The structure of the ocular surface comprises the adnexa, tear film, lacrimal gland, meibomian glands, cornea, conjunctiva, and eyelids (Craig et al., 2017). It protects the eyes from bacteria and chemicals, also becoming an obstacle to drug permeability. The blink reflex and tear turnover rate are the main contributing factors to a short ocular retention time (Chhonker et al., 2015). To remedy this problem, eye drops need to be frequently used to improve the therapeutic effect. This frequent administration leads to excess eye drops draining into the lacrimal duct and absorbed into the nasal mucosa, which may cause serious side effects (Yuan et al., 2012; Lin et al., 2019). Besides, the requirement of multiple administrations, in turn, may cause patients to become noncompliant.

Therefore, there have been many researchers devoted to investigating various ophthalmic drug delivery systems (DDSs) to optimize the delivery mechanism of the drug (Dave et al., 2021; Tsao et al., 2021). An ideal ophthalmic DDS is expected to have properties including stimuli responsiveness, ocular biocompatibility, and ocular biodegradability (Nguyen and Lai, 2020). Likewise, there are many diseases in ophthalmology which require long-period administration, such as severe dry eye and glaucoma. Ocular DDSs are promising in this field according to recent research studies. Hollow mesoporous ceria nanoparticles loading Y-27632 were synthesized to control high intraocular pressure owing to its ability of sustained release (Luo et al., 2021). The DDS of ophthalmology has also been used to improve bioavailability. Kumari et al. (2021) developed a lipophile carrier using cholesterol-Labrafac^TM^ to deliver dexamethasone. It is found that this formulation had high internalization capacity for corneal epithelial cell and showed superior ability to reduce inflammatory factor expression, such as TNF-α, MMP-9, and IL-6, suggesting that it could be a viable choice to treat dry eye. Han et al. (2022) constructed a temperature-sensitive tacrolimus (FK506) gel to treat dry eye. It was made of monofunctional, polyhedral, oligomeric silsesquioxane and exhibited outstanding mucoadhesive ability. Besides, it could more effectively treat dry eye than other FK506 formulations in a murine model. Carbonized nano-gels showed superior antibacterial and antioxidant effects on keratitis caused by *S. aureus*, attributing to its ability of corneal penetration *via* opening epithelial tight junction (Lin et al., 2021). Axitinib nano-wafer contained arrays of nano-reservoirs loading drugs. It was proven that this ocular DDS could realize slow release to increase pre-ocular retention time and effective inhibition of corneal neovascularization in a lower dosing frequency (Yuan et al., 2015).

Dry eye is a prevalent and multifactor-induced autoimmune disease of the ocular surface. The characteristic symptoms are as follows: discomfort, visual disturbance, and tear film instability; the disease can damage the ocular surface (Workshop, 2007; Chen et al., 2020). Immunoregulation includes two parts, and they are in homeostasis in the normal state. One is immunosuppression including CD4^+^ regulatory T cells (Treg) that restrict and suppress excessive inflammation-like autoimmunity. The other is pro-inflammatory, including Th1, Th2, and Th17. However, in dry eye, the regulatory function of Treg is compromised (Chen et al., 2013). More and more evidences from experiments and clinics show that Th17-mediated pathogenesis plays a primary role in persistent ocular surface inflammation (Chen and Dana, 2021). Continued inflammation is the primary pathogenesis of dry eye (Wei and Asbell, 2014). Dry eye has a global prevalence of 5%–50%, making it a widespread problem (Stapleton et al., 2017). Using tear substitutes to relieve ocular discomfort is the primary treatment of the condition. Immune modulators such as cyclosporine A and FK506 are also medications to treat dry eye (Valim et al., 2015). FK506 is a potent macrolide immunosuppressant. As a hydrophobic drug, it would penetrate the cell membrane and then interact with FK506-binding protein-12 to regulate the activity of the calmodulin-dependent protein phosphatase. Calcineurin inhibition could inhibit the release of cytokines by the blockage of T cell activation and differentiation (Rusnak and Mertz, 2000). Compared with cyclosporine A, it is more effective and causes more minor irritation to the surrounding tissue, so FK506 is increasingly used in the clinical setting (Siegl et al., 2019). However, the narrow therapeutic window for FK506 raises concerns for its use clinically.

Lipids are the composition of the tear film and can slow evaporation of the liquid layer, so a supplement of lipids also can relieve the clinical manifestations of dry eye. Due to this, nanoscale DDSs, such as liposomes, have become increasingly of interest to ophthalmologists. Liposomes, primarily constituting phospholipids, are a good choice of the delivery system owing to their outstanding biocompatibility, feasibility, and tenability. They are spherical vesicles with a hydrophilic core and lipidic bilayers; they have also been proven to increase the solubility of both hydrophilic and hydrophobic drugs (Kayser et al., 2005; Lopez-Cano et al., 2021). A substantial body of studies shows that lipid constitution, particle size, and zeta potential affect delivery efficiency (Inokuchi et al., 2010; Schäfer et al., 2010). Positively charged or larger liposomes exhibit extended ocular residence times in and around the injection site during subconjunctival administration, while neutral, negatively charged, or small liposomes are rich in the limbus (Chaw et al., 2020).

Cationic liposomes can conjunct with negatively charged sulfated proteoglycans in the cell membrane, preventing rapid clearance and encouraging integration (Steffes et al., 2017; Han et al., 2020b). Many studies have demonstrated that positively charged nanoparticles can modulate cell membrane potential. The depolarization of the cell membrane could prompt cell drug uptake, but it is detrimental to cell proliferation. The extent of membrane potential change is concentration-dependent (Arvizo et al., 2010; Kim et al., 2013). The eye is a suitable organ for cationic liposome eye drops. First, the cornea is regarded as a relatively immuno-privileged site; this is attributed to the absence of both blood and lymphatic vessels (Kumar and Kumar, 2014). Second, the conjunctival sac is a small lacuna that would not contain many liposomes. In addition, blinking helps form a tear film on the cornea, and this efficiently redistributes free cationic liposomes across the ocular surface. Furthermore, the ocular surface is an anionic environment derived from a mucin layer, and this predisposes the surface to attract cationic liposomes electrostatically (Lin et al., 2019; Han et al., 2020a), which increases the pre-ocular time of liposomes. There are two main approaches to generate liposomes with positive zeta potential (Lin et al., 2019). One is to utilize cationic phospholipids to prepare the liposomes, such as (2,3-dioleoyloxy-propyl)-trimethylammonium (DOTAP). The other is to modify the liposomes with positive substances, such as poly-L-lysine (PLL), chitosan, and polyethyleneimine (PEI) (Schäfer et al., 2010; Chaiyasan et al., 2013; Sasaki et al., 2013). Due to its adjuvanticity, cationic lipids are used to carry therapeutic molecules, such as antigens, in the form of liposomes (Farhadi et al., 2021). DOTAP is a synthetic lipid widely used *in vitro* and *in vivo* (Firouzmand et al., 2013). It was reported that liposomes containing DOTAP and 1,2-dioleoyl-sn-glycero-3-phosphoethanolamine (DOPE) could activate the immune responses of Th1 and Th2 cells in mice (Nikpoor et al., 2019).

Herein, this study aimed to develop a cationic FK506 liposome with DOTAP, so that it would have increased interactions with the ocular surface, improving ocular retention time. This treatment may not only alleviate the ocular inflammatory response to dry eyes but also prompt the bioavailability of eye drops. It would reduce the administration time of the treatment, reduce the risk of adverse events, and improve patient compliance. The chemical and physical characteristics of the FK506 liposomes, including particle size, zeta potential, and entrapment efficiency, were assessed. The efficacy of the cationic liposomes for dry eye treatment was evaluated both *in vitro* and *in vivo*.

## 2 Materials and Methods

### 2.1 Materials

DOTAP, DOPE, cholesterol, 2,7-dichlorodi -hydrofluorescein diacetate (DCFH-DA), fluorescein isothiocyanate (FITC), 4’,6-diamidino-2-phenylindole (DAPI), and benzalkonium chloride were purchased from Sigma. 1,2-distearoyl-sn-glycero-3-phosphoethanolamine-N-[amino(poly(ethylene glycol))2000] (DSPE-PEG2000) and FITC- DSPE-PEG2000 were purchased from Yuan Ye Biological Company. FK506 was purchased from MedChemExpress. Sodium dodecyl sulfate was purchased from Sangon Biotech. 1X phosphate buffer solution (PBS) was purchased from Biosharp. Commercial FK506 eye drops were purchased from SENJU (FK506 concentration: .1%), and it was a white turbid liquid. Hylo-COMOD was a commercial artificial tear whose first ingredient listed .1% sodium hyaluronate, and it was purchased from URSA PHARM Arzneimittel DmbH. The chemicals and solvents used for mass spectrum–high-performance liquid chromatography (MS-HPLC) and HPLC were of HPLC grade, while the others were of analytical grade.

### 2.2 Synthesis of FK506 Liposome and FITC-Labeled FK506 Liposome

The FK506 liposomes were prepared using the thin film hydrated dispersion method. Five mg DOTAP, 5.3 mg DOPE (DOTAP: DOPE = 1:1 in molar rate), 5 mg DSPE-PEG2000, 2.77 mg cholesterol, and 2 mg FK506 were dissolved in 6 ml of trichloromethane in a round-bottom flask. A rotary evaporator was used to remove the organic solvent in a 40°C water bath under a .06–.08 MPa vacuum; the rotation speed was kept at a rate of 100 rpm. Then, solvent traces were removed by keeping the solution in a 0.1-MPa vacuum for 1 h at 100 rpm. The resultant dried lipid film was hydrated with 10 ml 1X PBS at 40°C for 20 min until it formed a homogenous emulsion. Last, the final product was obtained by squeezing the liposomes through a 0.4-μm pore-size polycarbonate film (Whatman, Life Science) to decrease the particle size at room temperature. The liposomes were stored at 4°C until use.

The FITC-labeled FK506 liposome was synthesized following the abovementioned steps, except using FITC-labeled DSPE-PEG2000 to replace DSPE-PEG2000.

### 2.3 Particle Size and Zeta Potential of the FK506 Liposome

Particle size, zeta potential, and polydispersity index (PDI) of the liposomes were measured by dynamic light scattering (DLS) conducted on a particle size analyzer (Zetasizer nano-ZSE, Malvern). The liposomes were diluted with ultrapure water at a ratio of 1:100 in advance. Every sample was tested three times.

### 2.4 Transmission Electron Microscopy

The morphology of the liposomes was analyzed using negative staining TEM (Tecnai G2 spirit, Thermo FEI). Ten μl of FK506 liposomes was dropped onto a 300-mesh carbon-coated copper grid. Excess liposome solution was stained before 1 μl 2% uranium acetate was added for 60s. The uranium acetate was then blotted, and another 1 μl drop of 2% uranium acetate was added; this process was repeated three times. The samples were then dried and examined at 120 kV.

### 2.5 Entrapment Efficiency and Loading Level

The entrapment efficiency was determined by ultrafiltration. Freshly prepared FK506 liposomes were added to the ultrafiltration centrifugal tube (Merck Millipore, 10,000 NMWL) and centrifuged at 3,000 g at 4°C for 60 min. The filtered liquid, which contained free FK506, was collected, then lyophilized for 24 h, and redissolved with .6 ml methyl alcohol. The concentration of FK506 in the filtered liquid was determined by HPLC. Entrapment efficiency was calculated by the following formula:

Entrapment efficiency(%)=total mass of added FK506−the mass of free FK506 total mass of added FK506×100%. (1)

Product loss was not considered in the preparation process, where the mass of free FK506 stands for the amount of FK506 in the filtered liquid, and the total mass of added FK506 is the initial mass of FK506 added into trichloromethane.

The FK506 loading level was calculated by the following formula:

$$\mathrm{FK506}\;\mathrm{loading}\;\mathrm{level}\left(\%\right)=\frac{\mathrm{total}\;\mathrm{mass}\;\mathrm{of}\;\mathrm{FK506}\;\mathrm{encapsulated}\;\mathrm{in}\;\mathrm{liposomes}}{\mathrm{total}\;\mathrm{mass}\;\mathrm{of}\;\mathrm{FK506}\;\mathrm{liposomes}}\mathrm{\times 100\%}.$$ (2)

Total mass of FK506 encapsulated in liposomes equals total mass of added FK506 minus the mass of free FK506, and the total mass of the FK506 liposome equals 20.07 mg minus the mass of free FK506.

### 2.6 *In Vitro* Release Profile of FK506 Liposome

The release profile of the FK506 liposome was confirmed by dynamic dialysis. First, FK506 liposomes were freshly synthesized in accordance with the procedures mentioned above; following this, PBS containing .1% sodium dodecyl sulfate (SDS) was prepared to serve as a release medium under sink condition (Dai et al., 2013). Ten ml of the FK506 liposomes was pipetted into a preprocessed dialysis bag (Biosharp, 3,500 MW); this was placed in 30 ml of the release medium and stirred at 200 rpm at 37°C. At the pre-established time points (.5, 1, 2, 4, 6, 9, 12, 24, 48, 72, 120, and 168 h), 1 ml of the release medium was taken out before 1 ml of the new release medium was added to maintain the volume of the release medium. All the samples were collected in centrifuge tubes and lyophilized for 24 h. The resulting powders were dissolved in .4 ml of methyl alcohol and centrifuged at 10,000 g for 10 min. The supernatant was then collected, and the final concentration of FK506 was measured by HPLC.

### 2.7 HPLC of FK506 Determination

The quantitative analysis of FK506 in entrapment efficiency was determined by HPLC (Shimadzu, Japan), while the corneal concentration was determined using MS-HPLC (Agilent Technologies, USA). The experimental conditions of HPLC were as follows: The mobile phase comprised acetonitrile/10 mM ammonium formate solution (72/28, v/v). The flow rate was kept at 1.2 ml/min, and 20 μl of the samples was injected; the absorption was monitored at 210 nm. Chromatographic separation was carried out on an InfinityLab Poroshell 120 SB-C18 column (50 × 4.6 mm, 2.7 µm), and the column temperature was maintained at 60°C. The retention time of FK506 on this column was approximately 1.6 min; the peak area was calculated using the least square method, and the standard curve of the FK506 concentration peak was pictured. The regression equation was used to calculate the corresponding FK506 concentrations when the areas were known.

### 2.8 Human Corneal Epithelial Cell Cultivation

HCECs were purchased from ATCC and cultivated at an atmosphere of 95% air/5% CO_2_ at 37°C. The culture medium used was Dulbecco’s modified Eagle medium (DMEM) (Solarbio); this was supplemented with 10% fetal bovine serum (Life Technologies, Grand Island, NY), 4.5 mg/ml glucose, 100 U/ml penicillin, and 100 ug/ml streptomycin.

### 2.9 Cytotoxicity

Cytotoxicity was measured using a cell counting kit-8 (CCK-8) assay (Dojindo, Japan) according to the manufacturer’s protocol. HCECs were seeded into 96-well plates at a ratio of 5,000 cells per well with 100 μl culture medium. The plates were placed into a cell incubator to allow them to adhere for 24 h. The cell culture medium was removed, and the cells were washed three times. The cells were incubated with 100 μl DMEM, blank liposomes, FK506 liposomes, and commercial FK506 eye drops for 2, 4, and 6 h. FK506 concentration of FK506 liposomes was .2 mg/ml, as well as that of commercial FK506 eye drops. Subsequently, 10 μl of the CCK-8 solution was added into each well, and then the cells were re-incubated for 2 h. The optical density values were measured at 450 nm using a microplate reader (SpectraMax M5, Molecular Device), and cell viability was calculated. The optimum concentration of FK506 liposomes was also determined using CCK-8 assay. FK506 liposomes with a series of drug concentration gradients (.0025 mg/ml, .005 mg/ml, .01 mg/ml, .02 mg/ml, .05 mg/ml, .1 mg/ml, and .02 mg/ml) were tested, and cell viability was calculated.

### 2.10 Cell Uptake

1*10^5^ HCECs were seeded into confocal dishes and adhered to for 24 h, the culture medium was then removed, and the cells were washed three times. Hundred μl of FK506 liposome labeled with FITC was co-incubated with cells for 2 h with 100 μl DMEM in the cell incubator. The excess liposomes and DMEM were removed, and the cells were washed three times. The nuclei of the HCECs were stained by DAPI. Confocal microscopy (A1 Ti, Nikon) was used to show the position of FK506 liposomes in cells.

### 2.11 Dry Eye Cell Model

The cell models of dry eye were established as follows: HCECs were seeded into 12-well plates at a density of 10^5^ per well and cultivated in the conditions mentioned above. After 24 h, the culture medium was removed, and 200 μM of hydrogen peroxide (H_2_O_2_) was added, and the cells were incubated for 1 h (Li et al., 2019).

### 2.12 The Efficiency of Reactive Oxygen Species Inhibition *In Vitro*

The HCECs were seeded at a density of 1.0 × 10^5^ cells per well with 100 μl culture medium. After 200 μM H_2_O_2_ treatment was carried out for 1 h, FK506 liposomes and commercial FK506 were added into each well. After co-incubation for 2 h, the cells were washed three times with PBS and stained with DCFH-DA for 30 min. The fluorescence intensity of intracellular ROS was measured by flow cytometry at 484 nm (excitation) and 520 nm (emission).

### 2.13 Dry Eye–Related Inflammatory Factor Determination

The processed HCECs were treated with 1 ml of TRIzol and collected. MRNA was collected, quantified, and reverse-transcribed in turn. Reverse transcription was performed using the PrimeScript™ RT-PCR Kit, followed by quantitative real-time polymerase chain reaction (qRT-PCR) using the SYBR® Premix Ex Taq™ Kit. All the experimental procedures were in accordance with the manufacturer’s protocols. The primer pairs were as follows: human GAPDH, 5′-ACA​ACT​TTG​GTA​TCG​TGG​AAG​G-3′ (forward) and 5′-GCC​ATC​ACG​CCA​CAG​TTT​C-3′ (reverse); human interleukin-1β (IL-1β), 5′-AGC​TAC​GAA​TCT​CCG​ACC​AC -3′ (forward) and 5′-CGT​TAT​CCC​ATG​TGT​CGA​AGA​A -3′ (reverse); and human interleukin-6 (IL-6), 5′- ACT​CAC​CTC​TTC​AGA​ACG​AAT​TG -3′ (forward) and 5′-CCA​TCT​TTG​GAA​GGT​TCA​GGT​TG -3′ (reverse).

### 2.14 Animal Model

Animal experiments were carried out after ethical approval (No. 2021057) and conformed to the ethical standards of The Second Affiliated Hospital of Zhejiang University. The animal models of dry eye were established as follows: New Zealand rabbits (2–2.5 kg, male) were obtained from the Zhejiang province medical science institute. They were housed in their own cages individually in an environment where the temperature was 20–26°C and the humidity was around 40%–70%. The rabbits were treated with 25 μl of .2% benzalkonium chloride twice a day in the interval of 12 h for 7 days according to a previously reported study (Tan et al., 2019). C57BL/6 mice (20–25 g, male) were purchased from Shanghai SLAC Animal Co., Ltd. The mice were housed in pathogen-free conditions as stipulated.

### 2.15 *In Vivo* Biocompatibility

The biocompatibility of FK506 liposomes was evaluated in the aspect of central corneal thickness (TOMEY anterior segment optical coherence tomography, Japan), endothelial cell density (TOMEY specular microscope EM-3000, Japan), corneal edema and conjunctival edema, and hyperemia (slit lamp microscope). Eighteen normal New Zealand rabbits were randomly put into three groups: the PBS group, the FK506 liposome group, and the artificial tear group. Each group had six rabbits. The rabbits in the PBS group were given PBS, those in the FK506 liposome group were treated with fresh FK506 liposome, and those in the artificial tear group were given Hylo-COMOD eye drops. All of them were administered twice a day (8 a.m. and 8 p.m.) at 50 μl. The abovementioned indicators were rigorously observed at day 0, day 7, and day 28. The anatomical structure of the cornea and conjunctiva was observed by H and E staining.

### 2.16 Ocular Irritation Test

The ocular irritation of FK506 liposomes was evaluated *via* the modified Draize test (Bhatta et al., 2012). The normal rabbits were treated with 20 μl of FK506 liposomes directly on the cornea in the right eye and 20 μl of PBS in the left eye. All of them were installed at 30-min interval for 6 h. Then, ophthalmological tests were performed to observe the level of irritation, including secretions, redness of conjunctiva, and corneal opacity and swelling. The observations were conducted at a 12-hour interval for 72 h.

### 2.17 Amount of FK506 Permeating Into the Cornea

The dry eye rabbits were treated with 50 μl of FK506 liposomes in the right eye and 50 μl of the commercial FK506 eye drops in the left eye. After 5, 30 min, 1, and 2 h, the rabbits were euthanized. The cornea was excised wholly and washed gently three times with normal saline. All samples were stored at −20°C. In addition, the amount of FK506 in the cornea was determined by MS-HPLC.

### 2.18 MS-HPLC of FK506 Determination

The experimental conditions of MS-HPLC were as follows: The cornea was cut into pieces and placed in centrifuge tubes. One ml of methyl alcohol was added along with 2 mm ceramic beads to prepare the cornea homogenate. All the samples were centrifuged at 10,000 g for 10 min, and the liquid supernatant was removed. The FK506 concentrations were measured utilizing MS-HPLC. The mobile phase comprised 0.1% formic acid/5 mM ammonium acetate in water (Solvent A) and acetonitrile (Solvent B), the flow rate was kept at 0.3 ml/min, and 20 μl of the samples was injected. Chromatographic separation was carried out on a Zorbax SB C18 column (150 × 2.1 mm, 3.5 µm). The gradient program was as follows: 80% of B for .5 min; kept at 95% of B for 4.5 min and finally, remained at 100% of B. MS was performed under 5 L/min gas flow at 325°C and 11 L/min sheath gas flow at 350°C. The ion spray voltages were 3,000 V and −3,500 V in the positive and negative ionization modes, respectively. The integral peak area of m/z 821.5>786.4 was used for quantitative analysis.

### 2.19 Retention Time in the Ocular Surface *In Vivo*

The pre-ocular retention time was assessed by *in vivo* fluorescence imaging. The C57BL/6 mice were anesthetized, and a drop of FITC-labeled FK506 liposomes and commercial FK506 eye drops mixed with FITC and FITC solutions (nearly 5 μl) was added to their eyes according to the group. The excitation wavelength was set at 488 nm, and the emission wavelength was 520 nm. After 5, 10, and 30 min, the fluorescence signal was collected and analyzed.

### 2.20 Effect of Alleviating Dry Eye Clinical Signs *In Vivo*

The 16 dry eye rabbits were assigned into four groups using a random number table, and they were as follows: the negative control group, positive control group, commercial FK506 eye drop group, and FK506 liposome group. Each group had four rabbits. The rabbits in the negative control group had no treatment or intervention. The rabbits in the positive group were given PBS after modeling of dry eye, commercial FK506 eye drop in the commercial FK506 eye drop group, whereas FK506 liposome in the FK506 liposome group. All of them were administrated twice a day (8 a.m. and 8 p.m.) at 50 μl.

To evaluate the clinical signs of dry eye, two ophthalmological tests were carried out on day 0, day 7, and day 9, including corneal fluorescein staining and TFBUT. Corneal fluorescein staining was carried out as follows: sodium fluorescein strips were wetted with a drop of normal saline and used to dye the conjunctiva of the lower eyelid; the rabbits blinked, and then corneal staining was observed and graded according to the SICCA OSS Group Scale. TFBUT was measured as follows: the rabbits were helped to blink several times to form a tear film in the ocular surface, and it started to time until the first dark point or crack appeared, and the abovementioned steps were repeated three times, and the average value was counted (Valim et al., 2015).

### 2.21 Corneal Epithelial Layer Thickness Measurement

The corneal epithelial layer thickness was measured using ImageJ software. The H and E–stained slides were photographed using a fluorescence microscope (Nikon DM4000) at 400 times magnification, and ten points were randomly chosen to measure the thickness. The mean value was calculated, and the difference was analyzed.

### 2.22 Enzyme-Linked Immunosorbent Assay and Hematoxylin–Eosin Staining

The cornea and conjunctiva of rabbits were collected following euthanasia on day 9. Part of these was fixed by 4% paraformaldehyde, embedded, and sectioned to observe corneal epithelial regeneration, conjunctival goblet cell density, and inflammation cell infiltration *via* H and E staining. The remaining tissue was kept in liquid nitrogen before being used to determine the level of inflammatory cytokines by ELISA. Inflammatory cytokines in the cornea and conjunctiva were extracted. The tissue was cut into pieces and further broken up using ultrasonication at 0°C. The resulting homogenate was centrifuged at 2,000 rpm for 20 min to collect the protein. The ELISA procedures were conducted according to the manufacturer’s protocol (Jiancheng Bioengineering institute, Nanjing, China).

### 2.23 Statistical Analysis

All the data in this article were reported as the mean ± standard deviation (SD). The statistical analyses performed were one-way ANOVA to calculate the significance of experimental data, except the analysis of FK506 concentration in the cornea and the comparison of relative corneal fluorescein score change between day 7 and day 9 using unpaired *t*-test (*p* < .05).

## 3 Results

### 3.1 Characterization of FK506 Liposomes

**FIGURE 1:**
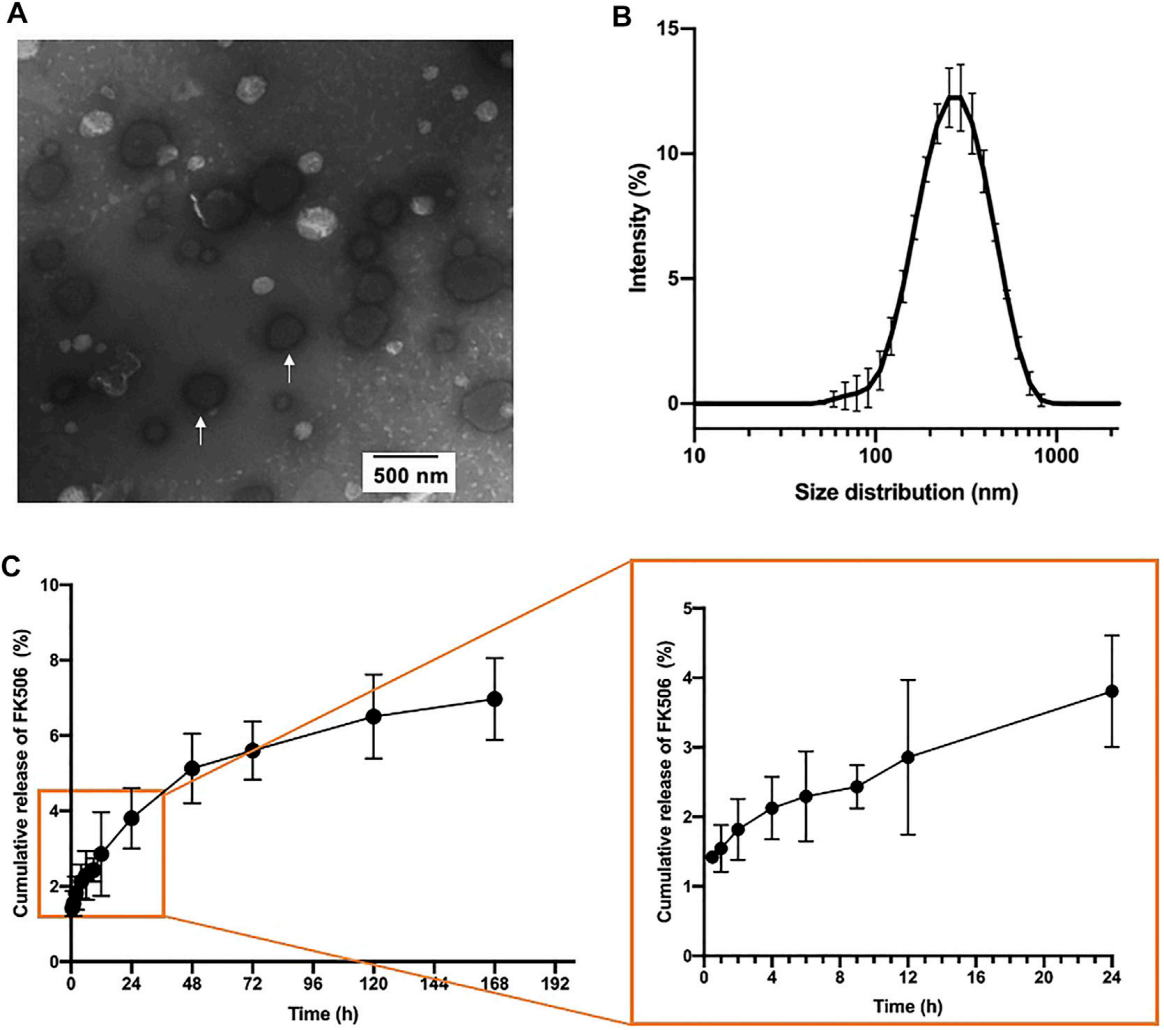
Physicochemical properties of FK506 liposomes. **(A)** Representative TEM image of FK506 liposomes (white arrow). **(B)** Size distribution of FK506 liposomes in PBS. (*n* = 3). **(C)** FK506 release profile *in vitro* in 7 days (right) and partial enlarged image (right). (*n* = 4).

The FK506 liposomes were synthesized using the classical thin-film hydration dispersion method (Wang et al., 2021; Wu et al., 2021; Zhong et al., 2021), as described in the *Materials and Methods*. The physical and chemical properties of the liposomes were investigated. The particle size of the FK506 liposomes was around 292 ± 7 nm, while the surface zeta potential was 31.1 ± 0.83 mV (Table 1). TEM showed that the morphology of the FK506 liposomes was spherical (Figure 1A), and the size distribution of the FK506-loaded liposomes was a little smaller in TEM than DLS. It might be attributed to the fact that DLS determined the hydrodynamic radius of nanoparticles. The entrapment efficiency of the FK506 liposomes was 90.11 ± 2.87%, and the FK506 loading level was around 9.07 ± 0.03%.

**TABLE 1:** Characterization of the FK506 liposome.

Sample	Particle size (nm)	Polydispersity index	Zeta potential (mV)	Entrapment efficiency (%)	FK506 loading (%)
FK506 liposomes	292 ± 7	0.27 ± 0.02	31.13 ± 0.83	90.11 ± 2.87	9.07 ± 0.03

The *in vitro* release profile of the FK506 liposome showed that FK506 liposome released FK506 slowly overall (Figure 1C). This formulation released the drug slowly in the first 48 h, and nearly 5.13 ± 0.92% FK506 was released in this period. There was an accumulative concentration of 6.97 ± 1.08% of total FK506 released until day 7.

### 3.2 *In Vitro* Cytocompatibility

**FIGURE 2:**
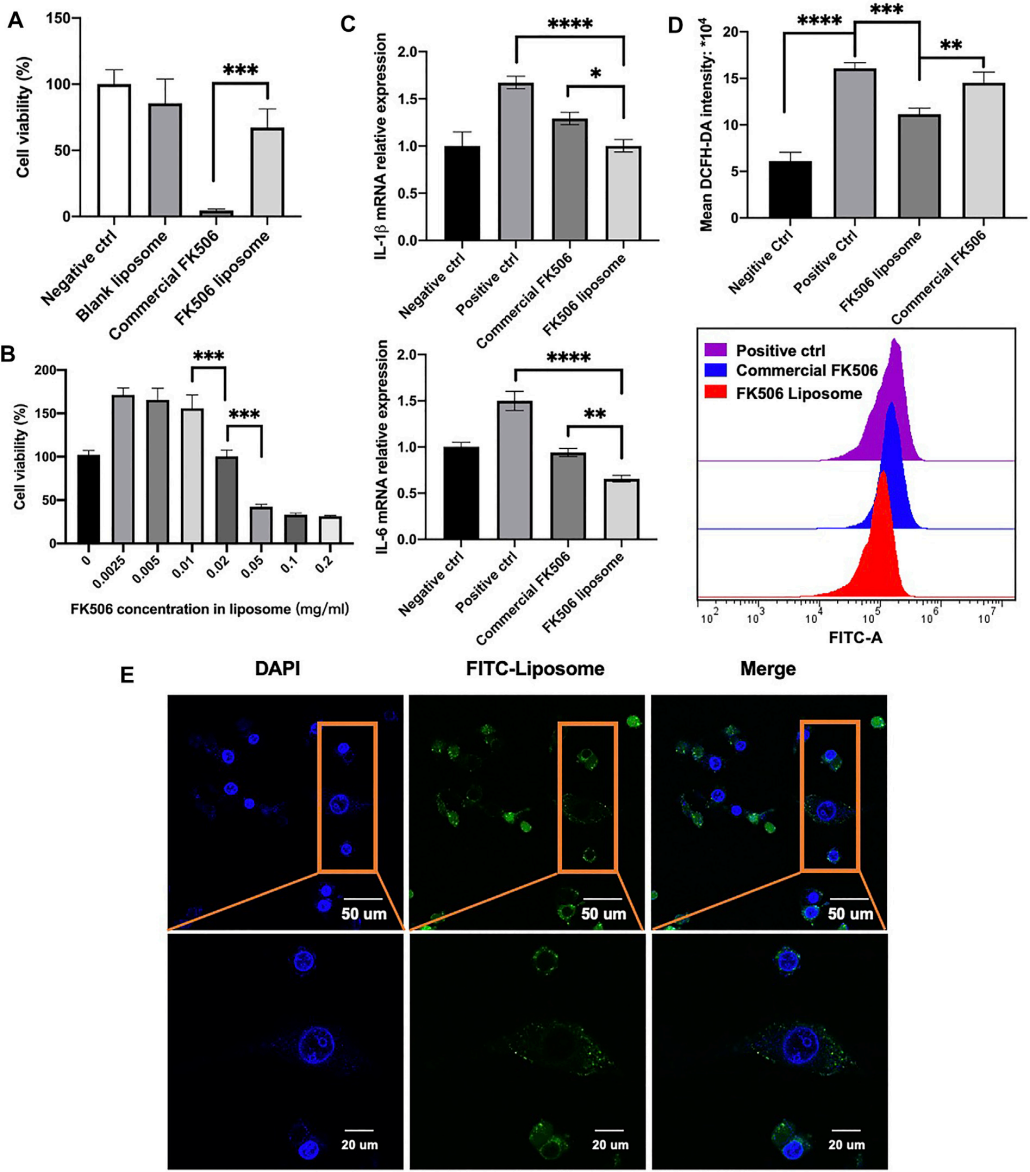
*In vitro* cytotoxicity test and efficiency evaluation on HCECs. **(A)** Cell viability test *via* CCK-8. HCECs were treated in a 0.2-mg/ml concentration of FK506 for 2 h (*n* = 5). **(B)** Cell viability test of a gradient of FK506 concentration in liposomes. HCECs were treated for 24 h (*n* = 4). **(C)** mRNA expression level of inflammation-related factors in the cornea and conjunctiva tissues after receiving different treatment. IL-1β is shown in the upper image (*n* = 3), and IL-6 is shown in the lower image (*n* = 3). **(D)** Flow cytometry analysis of ROS level (*n* = 3). *p < .05, **p < .01, and ***p < .001. **(E)** Co-localization of FK506 liposomes and HCECs. The cell nucleus is stained blue, and the FK506 liposome is stained green.

The positive charge of the liposome surface played a significant role in adhesion; however, it was also responsible for the cytotoxicity of cationic liposomes (Kim et al., 2013). Therefore, to evaluate the cytocompatibility of FK506 liposomes, the cell viability was measured using CCK-8. As shown in Figure 2A, the blank liposome group showed 85.54 ± 18.31% cell viability after 2-hour co-incubation with HCECs. In addition, there was no significant difference in viability between the blank liposome group and the negative control group. It suggested that the cationic liposomes did not exhibit apparent cytotoxicity. Furthermore, at the same FK506 concentration of 0.2 mg/ml, FK506-loaded liposomes were less detrimental to HCECs than commercial FK506 eye drops after 2 h. The cell viability was 67.17 ± 0.14% in the FK506-loaded liposome group and 4.38 ± 1.21% in the commercial FK506 eye drop group, and there was a statistically significant difference (*p* < .001). This result inferred that using liposomes as the drug delivery vehicle decreased drug toxicity.

To reduce the cytotoxicity further, a gradient of concentration test was then performed to determine the safe concentrations of the formulation *in vitro*. After 24-hour co-incubation, FK506 liposomes with a drug concentration below 0.02 mg/ml showed good cell viability (Figure 2B). Based on this result, .02 mg/ml FK506 concentration was chosen in the subsequent experiments *in vitro*.

### 3.3 FK506 Liposome Cellular Intake and Co-Localization *In Vitro*

The ability to enter cells was a prominent characteristic of cationic liposomes. It was important to deliver drugs (Takikawa et al., 2020). To investigate whether FK506 liposomes could enter cells, confocal microscopy was used. The nucleus was stained blue using DAPI, while FK506-loaded liposomes were stained green using FITC-labeled DSPE-PEG2000 (Figure 2E). After a 2-hour co-incubation with HCECs, FITC-labeled FK506 liposomes were shown to adhere to HCECs and were observed in the cytoplasm. This result implied that HCECs will uptake FK506 liposomes, facilitating the transfer of FK506 into cells.

### 3.4 The FK506 Liposome–Mediated Effect on Inflammatory Factor Expression and Reactive Oxygen Species Production *In Vitro*

Oxidative stress was pathogenesis of dry eye and was associated with its progression (Perez-Garmendia et al., 2020). ROS levels were evaluated using flow cytometry *via* the measurement of the mean intracellular fluorescence intensity. In Figure 2D, the mean ROS fluorescence intensity of the FK506 liposome group was 11.14 ± .65*10^4^; this result was significantly lower than that of the positive control group, which scored 16.07 ± .94*10^4^. It indicated that FK506 liposomes might reduce ROS generation following H_2_O_2_ stimulation. In addition, the FK506 liposome group had significantly lower ROS levels than the commercial FK506 eye drops group, which scored 14.52 ± 1.16*10^4^ (*p* < .001).

In terms of the dry eye–related expression of inflammatory factors, the levels of IL-1β and IL-6 were assessed using qRT-PCR. Compared to the positive control group, FK506 liposomes and commercial FK506 eye drops both downregulated the expression of IL-1β and IL-6. FK506 liposomes exhibited a more significant inhibitory effect on IL-1β (*p* < .05) and IL-6 (*p* < .01) expression than commercial eye drops. It was attributed to the fact that the liposomal FK506 dosing method allowed the drug to enter the cell easily, making it more efficient to decrease the expression of inflammatory factors, which was in accordance with previous studies (Nattika et al., 2014).

### 3.5 *In Vivo* Biocompatibility

**FIGURE 3:**
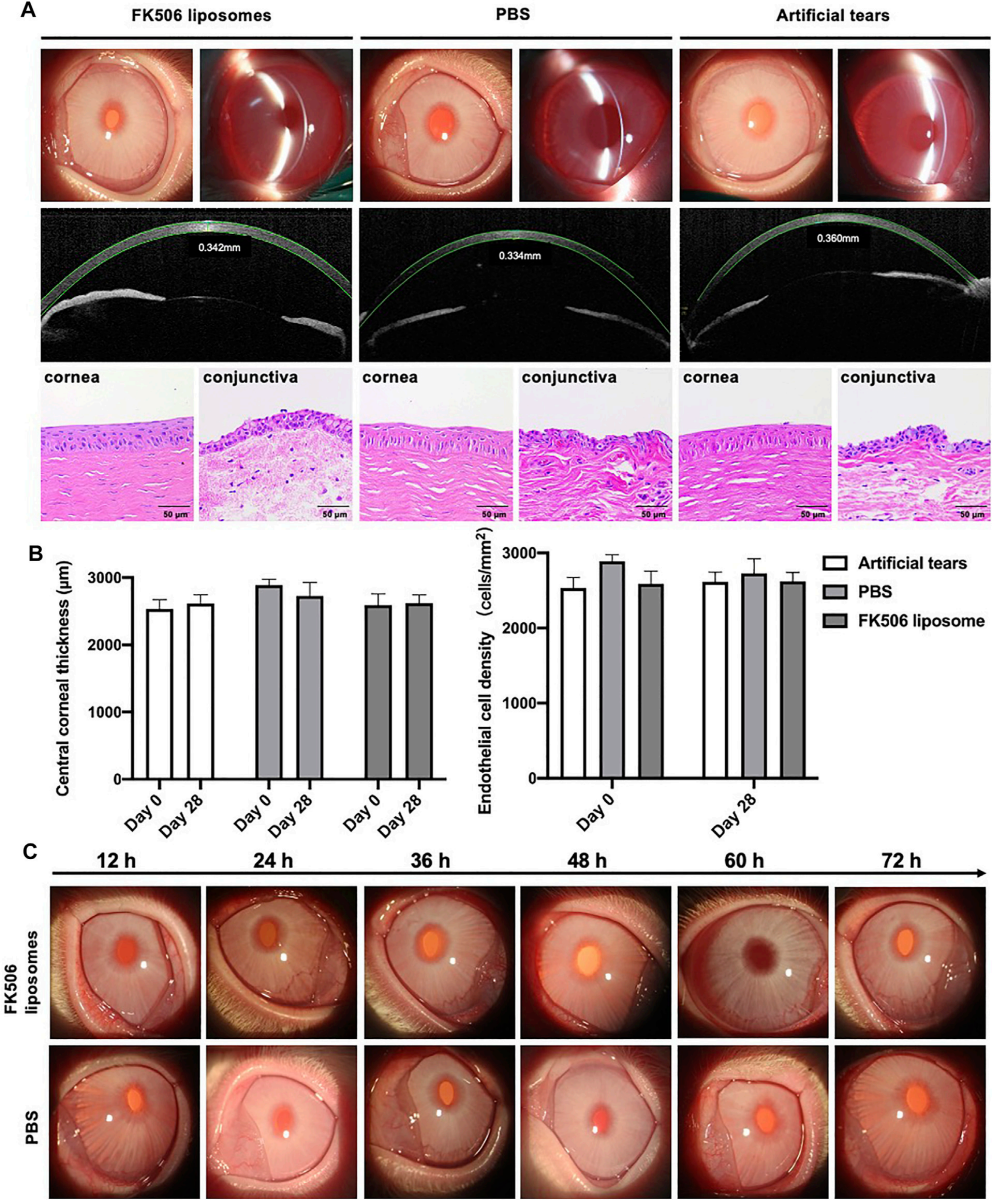
*In vivo* biocompatibility and ocular irritation evaluation of FK506 liposomes in New Zealand rabbits. **(A)** Upper image: anterior segment images observed by a slit-lamp microscope after the four-week administration of different eye drops. Middle image: corneal thickness measured by anterior segment optical coherence tomography after the 4-week administration of different eye drops. Lower image: H and E of the cornea (left) and conjunctiva (right) after the four-week administration of different eye drops. **(B)** Quantitative analysis of central corneal thickness (left) and endothelial cell density (right). **(C)** 72-hour observations of the ocular irritation test. The above-described images belonged to the FK506 liposome group, and the below images belonged to the PBS group.

The cornea is an extremely sensitive tissue due to dense coverage of nerve endings within the corneal epithelium, which protects the eyes from harmful substances and foreign objects (Lum et al., 2019). Once stimulated, tearing, blinking, and foreign body sensation in the eye occur; this progresses into pain, edema, optical congestion, and increased secretions. To assess the biocompatibility of FK506 liposomes, central corneal thickness and endothelial cell density were observed *in vivo*. The FK506 concentration in the FK506 liposome was 0.2 mg/ml. Both of the central corneal thickness and endothelial cell density remained stable after 28-day administration; there was no significant difference among the PBS group, commercial eye drop group, and FK506 liposome eye drops group. In addition to this, none of the groups presented obvious clinical signs of irritation. The cornea remained transparent throughout; there was no evidence of conjunctival congestion, edema, or a change to eye secretions during the observation period (Figure 3A). Moreover, H and E staining of the cornea and conjunctiva showed that there were no corneal epithelial defects, no corneal stromal edema, and no evidence of inflammatory cell infiltration (Figure 3B). Overall, it was concluded that FK506 liposome eye drops (FK506 concentration was 0.2 mg/ml) were compatible with the ocular surface and were safe for ophthalmic use.

### 3.6 Ocular Irritation Test

It was necessary to evaluate the ocular irritation *in vivo* because the surfactant is one of the materials to prepare FK506 liposomes. To study the ocular irritation of FK506 liposomes, the ocular irritation test was carried out. During the 72-hour observation, there were no signs of irritation after installing FK506 liposomes and PBS (Figure 3C). Specifically, there was no conjunctival redness, no secretions, and no corneal swelling. Thus, it was concluded that FK506 liposomes have no ocular irritation.

### 3.7 Ocular Retention Time and Amount of FK506 Permeating Into the Cornea

**FIGURE 4:**
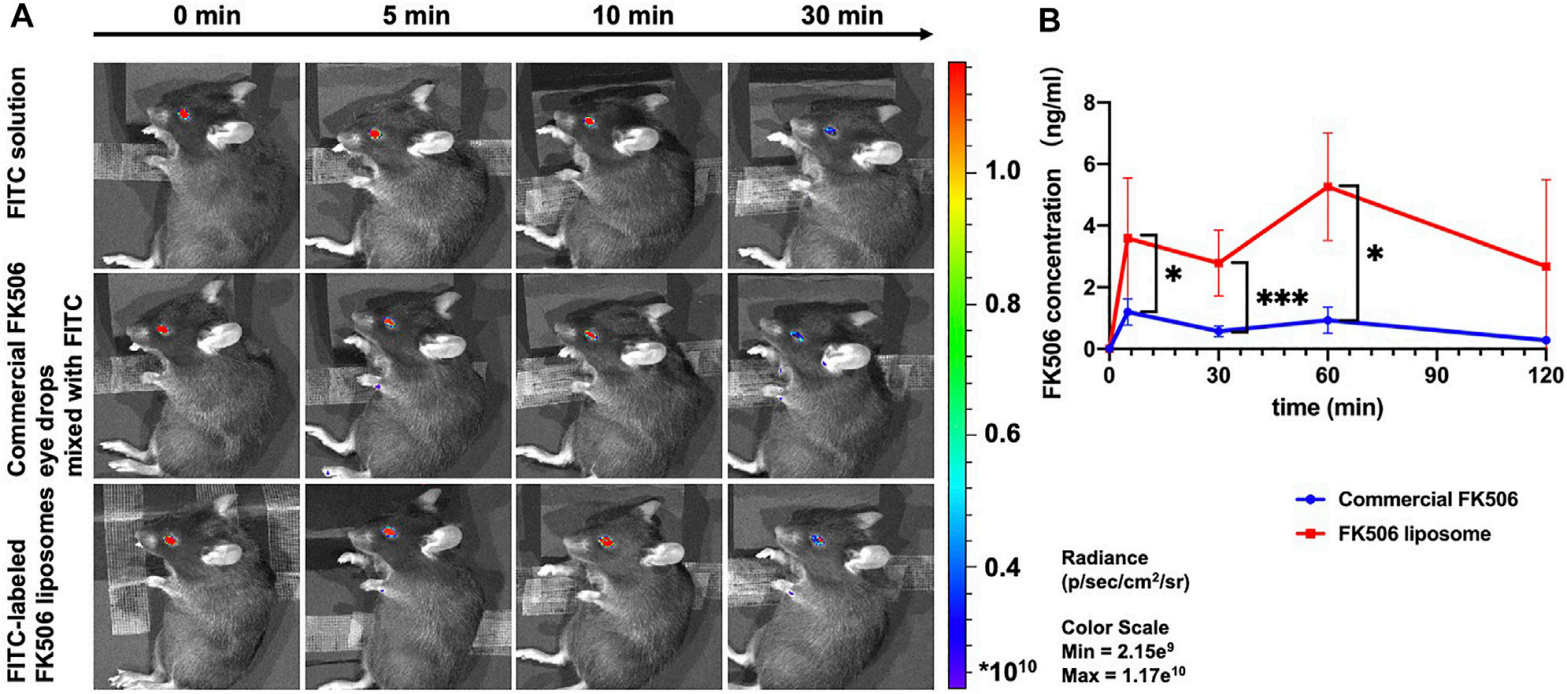
*In vivo* ocular retention evaluation of FK506 liposomes in C57BL/6 mice and trans-corneal FK506 amount change in New Zealand rabbits. **(A)** *In vivo* fluorescence images of FITC solution, commercial FK506 eye drops mixed with FITC, and FITC-labeled FK506 liposomes at 0, 5, 10, and 30 min. **(B)** *In vivo* trans-corneal FK506 amount changes of commercial FK506 eye drops (blue) and FK506 liposomes (red) after administration. Quantitative analysis of FK506 concentration in the cornea at 5, 30 min, 1 h, and 2 h. *P < .05 and **p < .01.

To investigate whether the use of FK506 liposomes would prolong retention time in the ocular surface, the fluorescence intensity following eye drop treatment was compared. Ten minutes after administration, there were similar fluorescence intensity levels among the three following groups, which are the FITC solution group, commercial FK506 eye drops mixed with FITC group, and FITC-labeled FK506 liposomes group (Figure 4A). However, only the group treated with FITC-labeled FK506 liposomes showed an intense signal after 30 min. Following this experiment, it can be concluded that FK506 liposomes can extend the ocular retention time.

In addition, the group treated with FK506 liposomes had a significantly higher FK506 concentration in the corneal tissue 1 h after administration than the group treated with commercial FK506 eye drops (Figure 4B (*p* < .01)). The FK506 concentration of the cornea in the FK506 liposome group was 3.59 ± 1.96 ng/ml, 2.79 ± 1.07 ng/ml, and 5.26 ± 1.75 ng/ml after 5, 30 min, and 1 h, respectively, while it was 1.20 ± .43 ng/ml, .57 ± .17 ng/ml, and .93 ± .43 ng/ml at the corresponding time points in the commercial FK506 eye drop group. It was in accordance with the ocular retention time, suggesting that the liposome was a promising drug delivery vehicle to facilitate FK506 penetration into the cornea.

### 3.8 The Effect on Alleviating Signs of Dry Eye *In Vivo*

As well as being a kind of macrolide antibiotic, FK506 is more commonly used in the suppression of the immune system. In ophthalmology, the primary use for FK506 eye drops was conjunctivitis catarrhalis aestiva. In addition, clinicians also prescribe FK506 for patients with severe dry eye or following corneal transplantation surgery.

**FIGURE 5:**
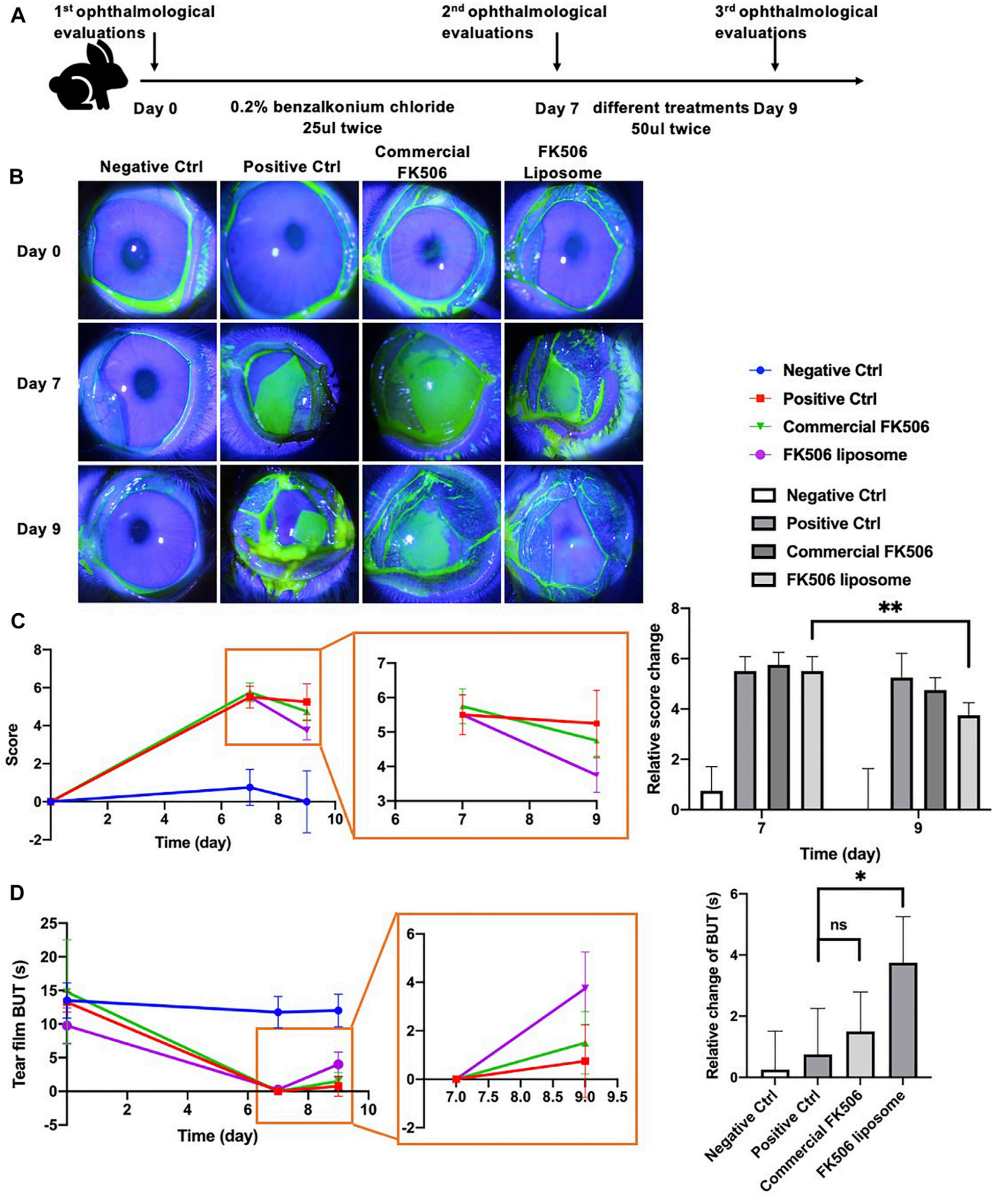
*In vivo* ophthalmological evaluations of FK506 liposomes in New Zealand rabbits with dry eye. **(A)** Flow diagram of the dry eye model and treatment plan. **(B)** Representative slit-lamp images of corneal fluorescein sodium staining at day 0, day 7, and day 9. **(C)** Relative corneal fluorescein staining score change at day 0, day 7, and day 9 (left); partial enlarged image (middle) and quantitative analysis of relative changes of score compared with baseline (day 0) (right). **(D)** Absolute BUT change at day 0, day 7, and day 9 (left); partial enlarged image (middle) and quantitative analysis of relative changes of BUT compared with day 7 (right). *p < .05 and **p < .01.

The scheme of the evaluation of the effect on alleviating signs of dry eye *in vivo* is shown in Figure 5A. As shown in Figure 5D, there was a sharp improvement to the tear film break up time (TFBUT) of the FK506 liposome group. Dry eye would interfere with the stability of the tear film and influence the health state of the ocular surface. TFBUT is an indicator of the stability of the tear film. The long TFBUT implies the stable tear film and mild dry eye. On the contrary, the short TFBUT hints at a brittle tear film and severe dry eye. Corneal fluorescein staining score could evaluate the health state of the ocular surface, especially the cornea. The appearance of fluorescein staining suggests that the integrity of the cell is compromised. The high corneal fluorescein score means corneal epithelial layer defect and severe dry eye (Workshop, 2007; Wolffsohn et al., 2017). Hence, TFBUT and corneal fluorescein staining score are indicators used to assess the severity of dry eye.

Compared with baseline (day 7), TFBUT increased by 3.57 ± 1.50 s when using FK506 liposomes; this was statistically significantly different to the 1.50 ± 1.29 s when using the commercial FK506 eye drops (*p* < .05). Notably, TFBUT was notably longer in the FK506 liposome group than in the positive control group at day 9 (*p* < .01). It showed that liposomes prepared as given above promote tear film stability. Similarly, corneal fluorescein staining levels were lower at day 9 in all groups than at day 7; the lowest levels were found in the FK506 liposome eye drop group. Statistical analysis showed a significant difference between day 7 and 9 (*p* < .01). By contrast, the commercial FK506 eye drop group showed no statistical difference between day 7 and 9 (Figures 5B,C). It indicated that there was a more rapid recovery of the corneal epithelial cell layer in the FK506 liposome eye drop group. FK506 liposomes not only cure epithelial cell injury but also prompt clinical sign alleviation.

### 3.9 The Effect on Prompting Corneal Epithelial Cell Layer Reconstruction *In Vivo*

**FIGURE 6:**
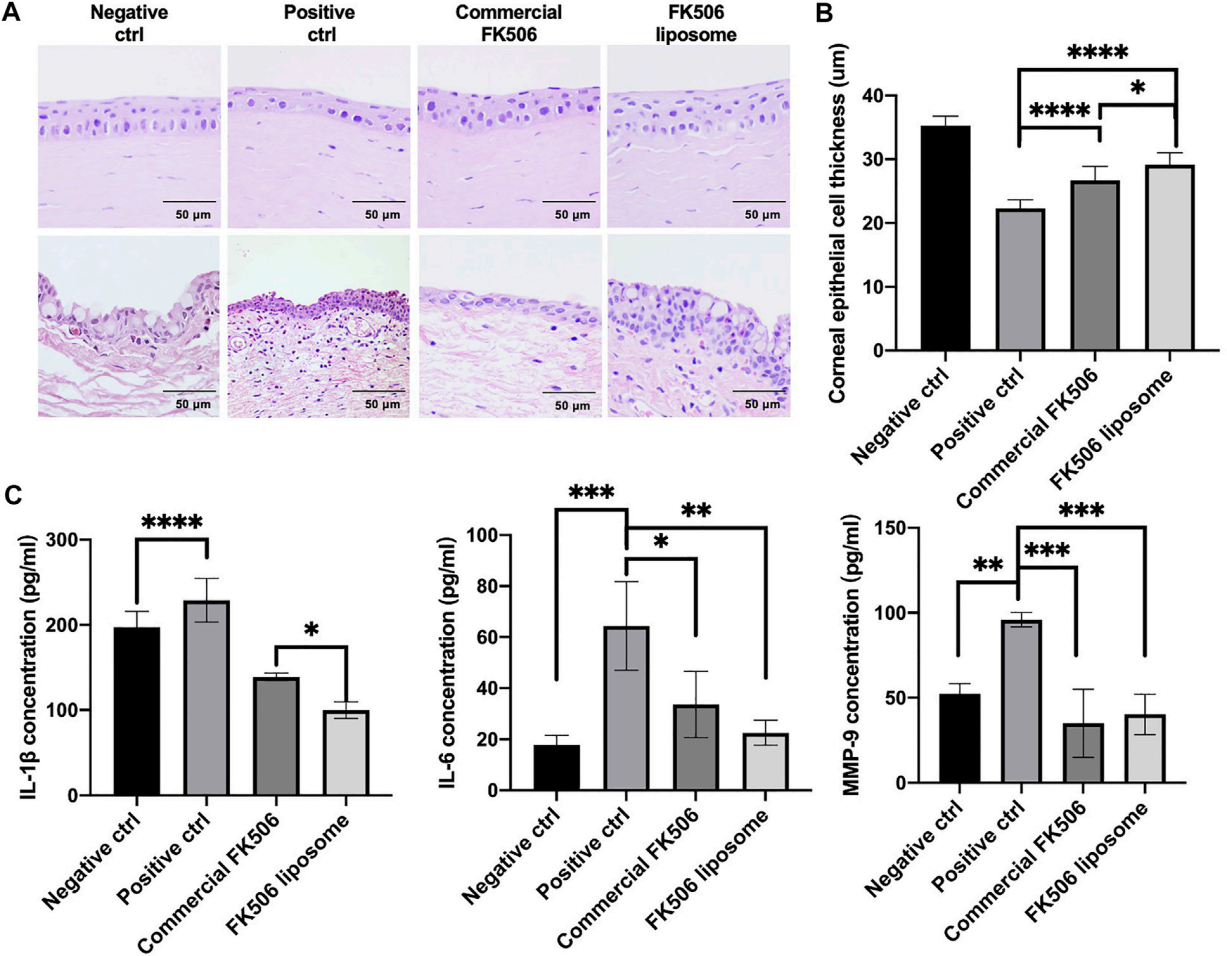
*In vivo* therapeutic effect evaluations on New Zealand rabbits with dry eye. **(A)** Representative H and E images of the cornea (upper) and conjunctiva (below) at day 9. **(B)** Quantitative analysis of corneal thickness at day 9. **(C)** Concentrations of inflammatory factors, including IL-1β, IL-6, and MMP-9, in the cornea and conjunctiva tissue. *p < .05, **p < .01, and ***p < .001.

Severe dry eye always has severe punctate erosions on the cornea, and fluorescein staining could help evaluate the range of corneal damage (Workshop, 2007). To assess the depth of corneal injury, the thickness of the corneal epithelial layer was measured. As is shown in Figure 6A, the reconstruction of the corneal epithelial cell layer varied widely from group to group at day 9. The commercial FK506 eye drop group and the FK506 liposome group had a more significant increase in thickness than the positive control group; in addition, the FK506 liposome group had a more considerable increase than the commercial FK506 eye drop group. The mean thickness of the epithelial layer was 22.31 ± 1.35 μm in the positive control group, 26.69 ± 2.18 μm in the commercial FK506 group, 29.14 ± 1.89 μm in the FK506 liposome group, and 35.28 ± 1.51 μm in the negative control group (Figure 6B). There was a significant difference between the positive control group and the FK506 liposome group (*p* < .0001), as well as the commercial FK506 eye drop group (*p* < .0001). Moreover, the difference between the FK506 liposome group and the commercial FK506 eye drop group was significant (*p* < .05).

### 3.10 The Effect on Decreasing the Expressions of Dry Eye–Related Inflammatory Factors *In Vivo*

To determine the effect of FK506 liposome eye drops on dry eye, the expression levels of dry eye–related inflammatory factors were determined *via* ELISA. As shown in Figure 6C, after treatment with .2% benzalkonium chloride, matrix metalloproteinase-9 (MMP-9), IL-1β, and IL-6 levels markedly increased in the cornea and conjunctiva. Following administration with FK506 liposomes, the concentration of IL-1β decreased to 99.91 ± 9.68 pg/ml, while the level in the commercial eye drops was 138.98 ± 4.32 pg/ml. This difference was statistically significant (*p* < .05). The level in the positive control group was 228.99 ± 25.59 pg/ml. Likewise, the expression levels of IL-6 and MMP-9 were downregulated in the FK506 liposome group at 22.58 ± 4.87 pg/ml and 40.26 ± 11.83 pg/ml, respectively. The concentration of IL-6 in the positive control group was 64.38 ± 17.35 pg/ml (*p* < .01) and the MMP-9 concentration was 95.95 ± 4.23 pg/ml (*p* < .001).

## 4 Discussion

To improve the ocular bioavailability of eye drops, a more efficient ocular delivery method would be developed; due to this, nanoscale delivery systems, such as liposomes, have become increasingly of interest. Cationic liposomes have been demonstrated to adhere to the anionic surface of cells and can be internalized (Steffes et al., 2017); this property has become universally exploited as drug delivery. The ocular surface is an ideal environment for cationic liposomes to use due to its relative immune privilege and the ability to interact with cationic liposomes. Hence, cationic liposomes were chosen to encapsulate FK506 and treat dry eye. In this study, cationic FK506 liposomes were proven to prolong ocular retention time and increase FK506 amount in the cornea; at the same time, this treatment alleviated the signs of dry eye and reduced the expression of inflammatory factors *in vitro* and *in vivo.*

Landucci et al. (2021) developed a thymoquinone-loaded liposome and found that it could reduce the toxicity at high dose in HCEC-2 cells. Huang et al. (2017) constructed the liposome encapsulating montmorillonite and betaxolol hydrochloride to treat glaucoma and found that liposomal formulation could effectively reduce cytotoxicity of betaxolol hydrochloride. Likewise, our study showed that the FK506 liposome could improve the cytocompatibility of FK506 and decrease the expression of inflammatory factors *in vitro*. It is a limitation that a long-term systemic safety assessment is absent in this manuscript because FK506 is an immunosuppressor and could cause side effects.

In term of the release profile of FK506 liposomes, it released FK506 slowly overall, which was in accordance with previous studies and was attributed to its hydrophobic property (Dai et al., 2013). FK506 is hydrophobic and has little solubility in water. SDS could help increase the solubility of FK506 so that it was added into the release medium to achieve solubility. SDS is a tenso-active agent, which has surface activity and critical micelle concentrations of the surfactant (Onwosi and Odibo, 2012). It could interact with liposomes and has an influence on the release profile (Zhang et al., 2011).

In terms of ocular bioavailability, Lin et al. (2019) found that compared with anionic nanoparticles and a commercial formula, drug amount in the cornea was highest in the administration group of cationic nanoparticles. It was in accordance with our study. Cationic FK506 liposomes had relatively higher FK506 amount in the cornea than commercial FK506 eye drops. According to the medication guide, commercial FK506 eye drops mainly consist of FK506 hydrate and .01% benzalkonium chloride. Benzalkonium chloride is a common preservative in ophthalmic preparations. Meloni et al. (2010) and Meloni et al. (2019) found that acute exposure of .01% benzalkonium chloride could reduce transepithelial electrical resistance value and increase the release of lactate dehydrogenase after 24-hour treatment and 16-hour recovery *in vitro*, but it was nontoxic after 24-hour treatment. Transepithelial electrical resistance value is an indicator of corneal barrier property, reflecting the epithelial thickness and integrity of tight junction. The release of lactate dehydrogenase correlates to the damage of the cellular membrane (Meloni et al., 2019). The corneal barrier affects the ocular bioavailability of eye drops. In the part of FK506 permeability into the cornea in our studies, we administered 50 μl of the commercial FK506 eye drops once and observed for 2 h. Therefore, it was referred that .01% benzalkonium chloride might have a tiny effect on promoting FK506 penetration into the cornea in the commercial FK506 eye drops. Positive charge on the surface of FK506 liposomes significantly improved permeability into the cornea.

Benzalkonium chloride is detrimental to corneal epithelium cells and could decrease the tear film break up time, especially in the patients with long-period administration (Meloni et al., 2019). However, Okahara and Kawazu (2013) found that when installing eye drops with .005% and .01% benzalkonium chloride, there was no obvious change on the ocular surface after 52-week administration. In our study, the administration time of commercial FK506 eye drops is short. Therefore, it is inferred that the influence of 0.01% benzalkonium chloride on the ocular surface was small. Tong et al. (2019) studied the influence of benzalkonium chloride on ROS production on HCECs and found that it was concentration-dependent. Namely, higher concentration of benzalkonium chloride induced higher level of ROS. Benzalkonium chloride at .02% increased ROS production, while at .001% induced very little ROS production. In our studies, based on the result of cytotoxicity of FK506 liposomes, .02 mg/ml FK506 concentration was chosen in the subsequent experiments *in vitro*. To achieve this, commercial FK506 eye drops with 1 mg/ml FK506 concentration were diluted. Meanwhile, the concentration of the preservative contained in this product was diluted to .002%. Therefore, benzalkonium chloride might have slight influence on ROS production of HCECs.

It is also of note that FK506 liposomes work with lower concentrations of FK506. In practice, commercial FK506 eye drops have a .1% FK506 concentration. Patients need to use regular drops twice a day, and this means that approximately the patients receive a .05 mg dose of FK506 at each treatment. In this study using FK506 liposomes, 50 μL of liposomes contained .01 mg of FK506, a fourfold reduction compared to commercial FK506 eye drops. It means that FK506 liposomes could relieve the clinical signs of dry eye in a lower concentration of the drug. It was attributed to the cationic charge of the liposome and surface zeta potential. With the cationic charge on the liposomal surface, liposomes loaded with FK506 could interact with HCECs and prolong ocular surface residence time. Nevertheless, when using regular eye drops, FK506 would be quickly cleared. To solve this problem, patients are required to install eye drops frequently to alleviate the clinical signs or use medications with high drug concentration. The FK506 concentration of commercial FK506 eye drops is higher than that of FK506 liposomes. Hence, commercial FK506 eye drops also alleviate the clinical signs of dry eye. In conclusion, both the commercial FK506 eye drops and FK506 liposomes boost epithelial cell regeneration and inhibit inflammatory reactions, while FK506 liposomes were significantly more effective. Using the liposome drops would increase ocular surface residence time, resulting in effective recovery of dry eye. Likewise, Gelfuso et al. (2020) found that liposomal formulations could enhance voriconazole penetration into the cornea, thus prompting recovery of fungal keratitis. Zhan et al. (2018) developed a tetrodotoxin-and-dexmedetomidine-loaded liposome to achieve long-acting ocular anesthesia. This liposome functionalized with Concanavalin A, which could be conjunct with corneal glycan moieties and prevent rapid clearance.

Above all, cationic FK506 liposomes could prolong precorneal time, prompt drug amount in the cornea, and relieve dry eye. The cationic liposome is a good choice of the ocular delivery system in ophthalmology.

## 5 Conclusion

This study reports that cationic liposomes encapsulating FK506 can prolong ocular retention time and increase FK506 amount in the cornea by interacting with the anionic ocular surface. Cationic FK506 liposomes could reduce ROS production in the cell model of dry eye following H_2_O_2_ treatment. In addition, it could decrease the expression of dry eye–related inflammatory factors *in vitro* and *in vivo*, as well as alleviate the signs of dry eye. Taken together, this study constructs a new delivery method for FK506 eye drops that have increased ocular bioavailability compared to standard treatments.

## Data Availability

The original contributions presented in the study are included in the article/Supplementary Material; further inquiries can be directed to the corresponding authors.
